# Variable echo time imaging for detecting the short T2* components of the sciatic nerve: a validation study

**DOI:** 10.1007/s10334-020-00886-w

**Published:** 2020-09-22

**Authors:** Paolo Florent Felisaz, Eugenio Belatti, Xeni Deligianni, Niels Bergsland, Francesco Santini, Matteo Paoletti, Francesca Solazzo, Giancarlo Germani, Andrea Cortese, Elisa Vegezzi, Oliver Bieri, Stefano Bastianello, Anna Pichiecchio

**Affiliations:** 1 Department of Neuroradiology, IRCCS Mondino Foundation, Pavia, Italy; 2 Department of Radiology, Desio Hospital, ASST Monza, Desio, Italy; 3 Department of Radiology, Division of Radiological Physics, University Hospital Basel, Basel, Switzerland; 4 Department of Biomedical Engineering, University of Basel, Allschwil, Switzerland; 5 Department of Neurology, Buffalo Neuroimaging Analysis Center, Jacobs School of Medicine and Biomedical Sciences, University at Buffalo, State University of New York, Buffalo, NY USA; 6 IRCCS, Fondazione Don Carlo Gnocchi, Milan, Italy; 7 Department of Brain and Behavioral Sciences, University of Pavia, Pavia, PV Italy; 8 Department for Neuromuscular Disease, UCL Queen Square Institute of Neurology and The National Hospital for Neurology, London, UK

**Keywords:** Magnetic resonance imaging, Peripheral nerves, Fibrosis, Validation study

## Abstract

**Objective:**

The aim of this study was to develop and validate an MRI protocol based on a variable echo time (vTE) sensitive to the short T2* components of the sciatic nerve.

**Materials and methods:**

15 healthy subjects (M/F: 9/6; age: 21–62) were scanned at 3T targeting the sciatic nerve at the thigh bilaterally, using a dual echo variable echo time (vTE) sequence (based on a spoiled gradient echo acquisition) with echo times of 0.98/5.37 ms. Apparent T2* (aT2*) values of the sciatic nerves were calculated with a mono-exponential fit and used for data comparison.

**Results:**

There were no significant differences in aT2* related to side, sex, age, and BMI, even though small differences for side were reported. Good-to-excellent repeatability and reproducibility were found for geometry of ROIs (Dice indices: intra-rater 0.68–0.7; inter-rater 0.70–0.72) and the related aT2* measures (intra-inter reader ICC 0.95–0.97; 0.66–0.85) from two different operators. Side-related signal-to-noise-ratio non-significant differences were reported, while contrast-to-noise-ratio measures were excellent both for side and echo.

**Discussion:**

Our study introduces a novel MR sequence sensitive to the short T2* components of the sciatic nerve and may be used for the study of peripheral nerve disorders.

## Introduction

MR neurography has gained increasing interest for the study of nerves in different conditions and new biomarkers sensitive to pre-clinically evident nerve damage are emerging [1, 2]. Compared to muscles in which degenerative changes are readily depicted as fat infiltration [3, 4], nerve degeneration is not commonly studied with MRI. The possibility to detect both early inflammation and chronic dystrophic changes could help to expand the role of MR neurography and its applicability to neuromuscular diseases in which the nerve is primarily concerned.

MR neurography protocols typically comprise conventional sequences that can detect signal from the long T2 components of nerves, represented by the nerve fascicles and the inter-fascicular fat. Turbo spin echo STIR, or T2-weighted fat-saturated and T1-weighted sequences are used to detect nerve damage, demonstrated as fascicles and total nerve area enlargement, increase of the overall T2 signal and the loss of the typical fascicular pattern. Quantitative MRI techniques, such as ADC maps and tractography, apparent-T2 maps, and magnetization transfer ratio (MTR) [5–7] have been suggested to be sensitive to tissue composition non-detectable with the resolution of conventional MRI. Quantitative MR neurography biomarkers are being used for the study of amyloid neuropathy, diabetic neuropathy and chronic inflammatory demyelinating polyneuropathy (CIDP) [8–12].

Peripheral nerves are complex structures made of different connective tissues that envelop the bundles of nerve fibers, including the endoneurium, perineurium, and epineurium [13]. All these connective layers provide structural support and trophic function, and may be altered in several pathological conditions. For instance in diabetic neuropathy and CIDP, the normal morphometry of the nerves is subverted, with changes in nerve diameter, edema, fascicles swelling or atrophy and variation of ratio between the fascicles and the epineurium [14, 15]. Imaging the collagenous connective tissue is technically challenging with MRI due to its intrinsic nature, which is characterized by short and ultrashort T2 components, typically producing little or no signal in the conventional sequences. In nerves, additional short T2 components arise from myelin making it even more difficult to reliably assess collagen and fibrosis [16, 17], but these precisely species could be interesting targets in chronic and demyelinating neuropathies.

Regarding the nerve in total, T2 values typically reported in the literature range from 45 to 70 ms at 3T. However, multiple water components with different relaxation times are present, most studies reporting three or more components ranging from 20 to 200 ms [18]. T2 is the transverse or spin–spin relaxation. In contrast to T2 that is an intrinsic property of the tissue, the T2* values are a combination of the T2 relaxation and external field inhomogeneities such as susceptibility variations, shimming, etc. Therefore, a reduction in T2* can be either due to a reduction in T2 or to other phenomena related to susceptibility. Eventually, a T2*-weighted acquisition [19] can potentially offer increased contrast of the nerves.

Different applications have been developed for the detection of short T2 components, including "Magic angle imaging" [20], zero-TE (ZTE) [21], ultrashort-TE (UTE) [22, 23], and short or variable echo time (vTE) sequences [24, 25]. Among them, vTE imaging is less technically demanding and can be implemented from standard gradient echoes sequences, employing conventional Cartesian encoding, asymmetric readout, and a variable TE minimized towards the center of the k space. TEs of 1 ms or less can be reached and it has been successful to highlight short T2* components within tendon and menisci [26]. A multi-echo approach allows to suppress the long T2* components by subtracting the longer echo to the shortest echo image and to calculate T2* maps [27]. However, since the decay of the signal in the nerve is very different from the background, even a dual echo acquisition can yield valuable information.

The aim of this work was to validate a vTE sequence for the study of the sciatic nerves in a sample of healthy subjects, for possible future application to populations with neuropathies. We propose calculating the apparent T2* as an extension to the principal of the apparent T2 (already successfully applied in by Kollmer et al. [2]). Our hypothesis is that it can potentially be sensitive to degenerative changes in peripheral nerves. We investigated differences in aT2* per thigh side and the potential influence of features such as age, sex, height, weight, and BMI, and nerve cross-sectional area (CSA). We assessed the repeatability and reproducibility of our methods, repeating the process by the same reader and two readers with different radiological experience. Signal-to-noise-ratio (SNR) and contrast-to-noise-ratio (CNR) were calculated for a subgroup of subjects.

## Materials and methods

### Study design and participants

The study was approved by the local ethics committee and all the participants provided informed consent. In total, 15 healthy volunteers were included: 10 participants (6 men and 4 women, mean age 24.4; range 21–26 years; weight 67.10 ± 8.12 kg; BMI 21.88 ± 1.37) were considered as the “young” group, whereas 5 volunteers with age over 45 years (3 men and 2 women; mean age: 55.8 years, range: 46–62 years; weight 67.20 ± 9.58 kg, BMI 21.93 ± 0.95) were considered as the “senior” group. A detailed medical history was obtained in addition to a targeted neurological examination. The subjects were excluded if they had either clinical evidence or past history of lumbar roots compression, peripheral polyneuropathy, diabetes mellitus, history of major trauma, or lower limb surgery.

### MR neurography

Imaging was performed on a 3T whole-body MRI scanner (Magnetom Skyra, Siemens Healthineers, Erlangen) with an 18-element received body array placed on the thighs to image the first third of the sciatic nerve. As a reference, we used a cutaneous tracker (vitamin E capsules) located 20 cm superiorly from the upper margin of the patella, and the volume of interest was placed with the last slice at the target.

The target of this study were the short T2* components of nerves such as the intraneural connective tissues and the myelin sheaths of the nerve fibers. Fan et al. reported appearance of contrast between nerve fascicles and their background (presumably composed of short T2* components) at about 9 ms. Reported values of T2* of single nerve fascicles and larger nerve areas range from 22 to 16 ms [28].

A 3D monopolar double-echo vTE spoiled gradient echo sequence [24, 25] was repeated twice for each subject to obtain images of both sciatic nerves. The first echo time was minimized by varying slightly the timing depending on the phase encoding and using asymmetric sampling (19% instead of 50%). For the first echo, the readout was asymmetric, while the second echo was fully sampled. Fast (1–1) binomial selective excitation was used to suppress the fat signal. The sequence parameters were as follows: TE1/TE2 = 0.98/5.37 ms, TR = 19 ms, flip angle = 10°, FOV = 290 × 236, voxel size = 0.6 × 0.6 × 5.0mm^3^, slices number: 12, bandwidth per pixel = 550 Hz/ Px, acquisition time = 5 min 40, and averages = 7. The longitudinal coverage of the sciatic nerve was of 6 cm. The subtraction technique was used as a method to suppress the long T2* components, enhance the visualization of short T2* tissue components, and calculate the apparent-T2* coefficient. The two echoes are sensitive to long T2* species; however, short T2* species are sampled mainly by the first echo and their signal reduces considerably at the time of the second echo. Subtraction image sets were reconstructed online [29]. As such, for each subject, we obtained 6 image sets made of 12 slices each (Echo 1, Echo 2 and Subtraction per side).

### Image processing and regions of interest (ROIs)

The sciatic nerve was manually delineated using a polygonal ROI mask with the software ITKSnap [30] (www.itksnap.org). ROIs were drawn on the Echo 2 image set because of the relatively better visualization of nerve contours and the absence of blurring due to symmetric readout. 10 out of the 12 slices of the image set were analyzed, discarding the two most extremes to avoid slab inhomogeneity. ROIs were then transferred to the corresponding Echo 1 image sets, as such identical ROIs were obtained for both Echo 1 and Echo 2 image sets. The entire process was performed by two readers with different clinical experience in MR neurography (PF 5 year experience, EB medical student) to assess interreader repeatability. One reader (EB) repeated the process twice (at T0 and at T1 after at least 48 h) on 5 random subjects, for intrareader repeatability. All cross-sectional areas (CSA) of the ROIs drown by Reader 2 were also recorded. Reader 2 drew additional round-shaped ROIs on all subjects on the background noise (i.e., the area outside the thighs) and on muscular tissue adjacent to nerve, aimed at signal-to-noise (SNR) and contrast-to-noise (CNR) measurements.

As an indicator of T2* differences, apparent-T2* (aT2*) was considered appropriate as a quantitative parameter for comparison between different sides and subjects. We calculated the aT2* values of the sciatic nerves from the two echoes image sets using the following equation on Matlab (ver. R2018b, The MathWorks Inc., Natick, Massachusetts, USA):

$$a{\text{T2}}^{*} = \frac{{ - \Delta {\text{TE}}}}{{\ln \frac{{i{\text{TE}}2}}{{i{\text{TE}}1}}}}.$$

Here, *i*TE2 is the signal intensity of the nerve ROI on Echo 2 image sets, *i*TE1 is the signal intensity of the nerve ROI on the Echo 1 image sets, and $\Delta TE$ is the difference of TE of Echo 1 and Echo 2.

### Statistical analysis

Statistical analysis was performed using Medcalc (MedCalc Software, Ostend, Belgium). Mean values of the aT2* measurements were calculated per each image-set. After assessing normal distribution of data, we used the paired Student's *t* test to evaluate aT2* differences per side, sex, and age. The Wilcoxon test was used for other non-normally distributed data such as the age- and sex-related per-side variability. A multivariable regression analysis was performed to evaluate the potential dependence of aT2* measures on variables such as weight, height, and BMI. Mean CSA values were also calculated per each sciatic nerve (nerve ROIs of Echo 1 and Echo 2 were identical). The *t* test was used to assess differences in CSA per side, age, and sex.

The intraclass correlation coefficient (ICC) was used for assessing intra- and interreader agreement for aT2* measurements (different measures are related to the definition of the ROIs**)** [31], and the Dice index was used for assessing geometric repeatability and reproducibility of the drawn ROIs among readers [32].

For SNR and CNR assessment, the following formulas were used: SNR = (mean value of the sciatic nerve signal/SD of the noise); CNR = (SNR (background muscle)-SNR(ROI of the sciatic nerve))/SNR(background muscle). Mean values of SNR and CNR per side and Echo 1 and Echo 2 image sets were calculated. Statistical tests were two-sided, with initial α level of significance defined at *p* < 0.05, and Bonferroni correction was used in case of multiple comparisons.

## Results

**Fig. 1:**
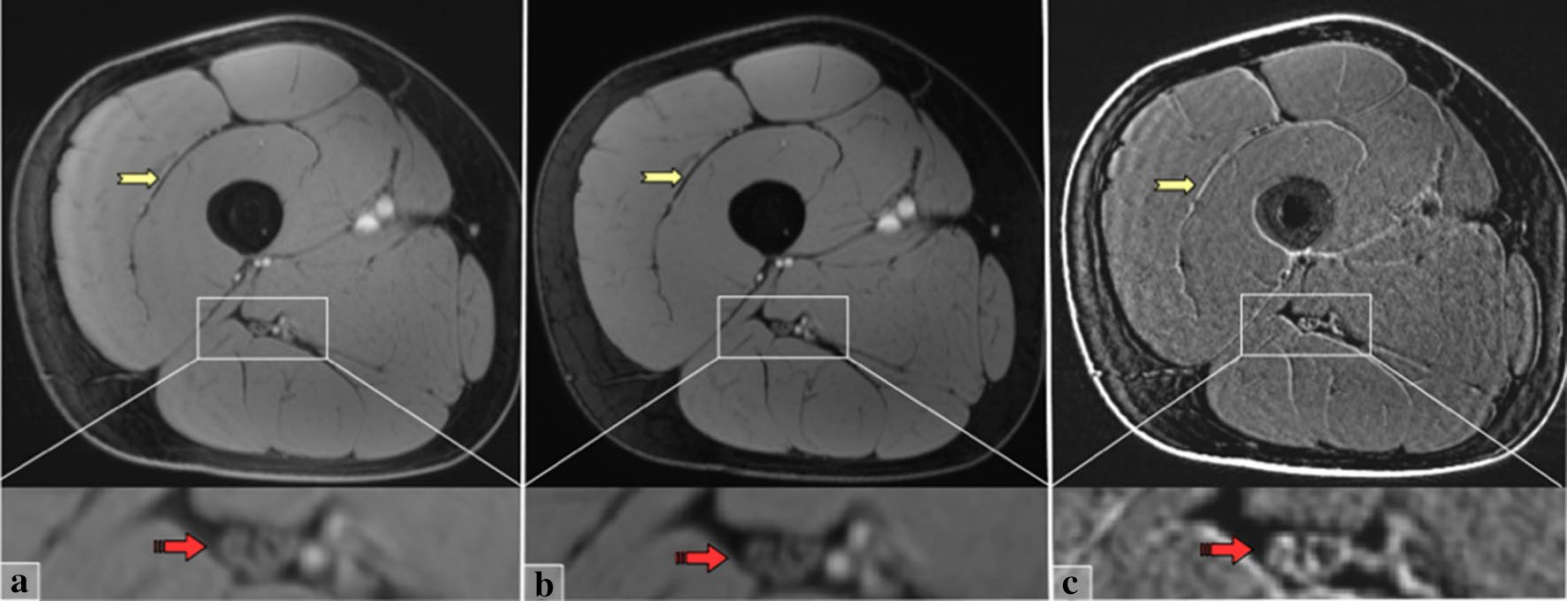
From left to right three axial sections of the right thigh, obtained with the vTE sequence. **a** Echo 1 with TE1 = 10.9 ms. **b** Echo 2 with TE2 = 5.37 ms. **c** Subtraction image. At the bottom, a zoom on the neurovascular fasciculus (white rectangle) visualizes the sciatic nerve. Image (**a**, **b**) has quite similar appearance; in **a,** short T2* components are present but masked by the longer T2* components; in **b,** short T2* components are completely decayed and only long T2* components are highlighted (both images are fat suppressed). In the subtraction image (**c**), the longer T2* components are suppressed and tissues made of short T2* components are highlighted. The nerve visualization (red arrow) changes in the Subtraction image (**c**): some of the fascicles disappear, others are encompassed in brighter areas. In muscles, tendons and fascial structures (yellow arrows) appear dark in figures (**a**, **b**), while in the subtraction image, they appear distinctly bright

Figure 1 shows a typical example of the vTE images. Spatial resolution and contrast are sufficient to reliably depict the sciatic nerve. Fat suppression is homogeneous to highlight the neuromuscular tissues of the thigh. Although the native Echo 1 and Echo 2 images are similar in appearance, the subtraction image effectively highlights the short T2* components by suppressing the other structures. Specifically, on the subtracted image, the nerve fascicles (mainly made of long T2* components) show reduced signal, whereas the inter-fascicular tissues (mainly made of connective fibrous tissue with shorter T2* components) appear brighter. This effect is even more evident in muscles, with the fascia and intramuscular tendons and septa standing out, and decreased signal of the muscle tissue (with longer T2*).

Table 1 reports the demographic data in the young and senior population, per each side. Only age was significantly different between the two groups. The multivariable regression analysis showed no significant influence of height, weight, nor BMI on aT2* measurements.

**Table 1:** Results of testing between the group of young versus senior volunteers: demographic variables of the population

Parameter	Group 1 (young)	Group 2 (senior)	*p* value (*t* Test)	Multivariable regression	
				Std error	*p* value
Sex (M/F)	6 / 4	3 / 2	N/A	N/A	N/A
Age	24.4 ± 1.58	55.8 ± 6.26	**0.0011**	N/A	N/A
Body weight (Kg)	67.1 ± 8.12	67.2 ± 9.58	0.902	1.94	0.475
Height (m)	1.75 ± 0.08	1.75 ± 0.11	0.854	144.77	0.462
BMI (Kg/m^2^)	21.88 ± 1.37	21.94 ± 0.95	0.859	5.74	0.447

Bonferroni correction for multiple testing: *α* value 0.05/6 = 0.008

Significant differences are given in bold

**Fig. 2:**
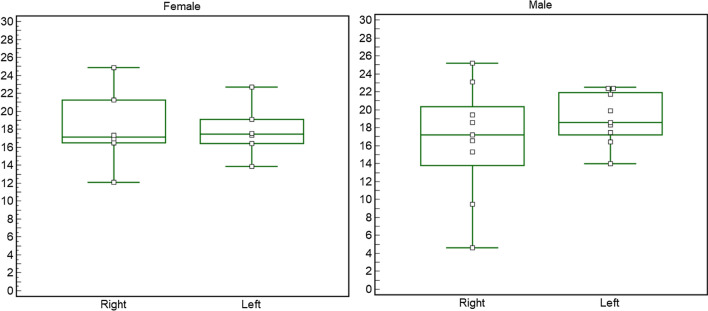
Graph representing the side differences obtained with the Wilcoxon test within the male group (nine subjects) and the female group (six subjects). The Wilcoxon test revealed no significant differences in side for aT2* (females *p* = 1.000; males *p*  = 0.300)

**Fig. 3:**
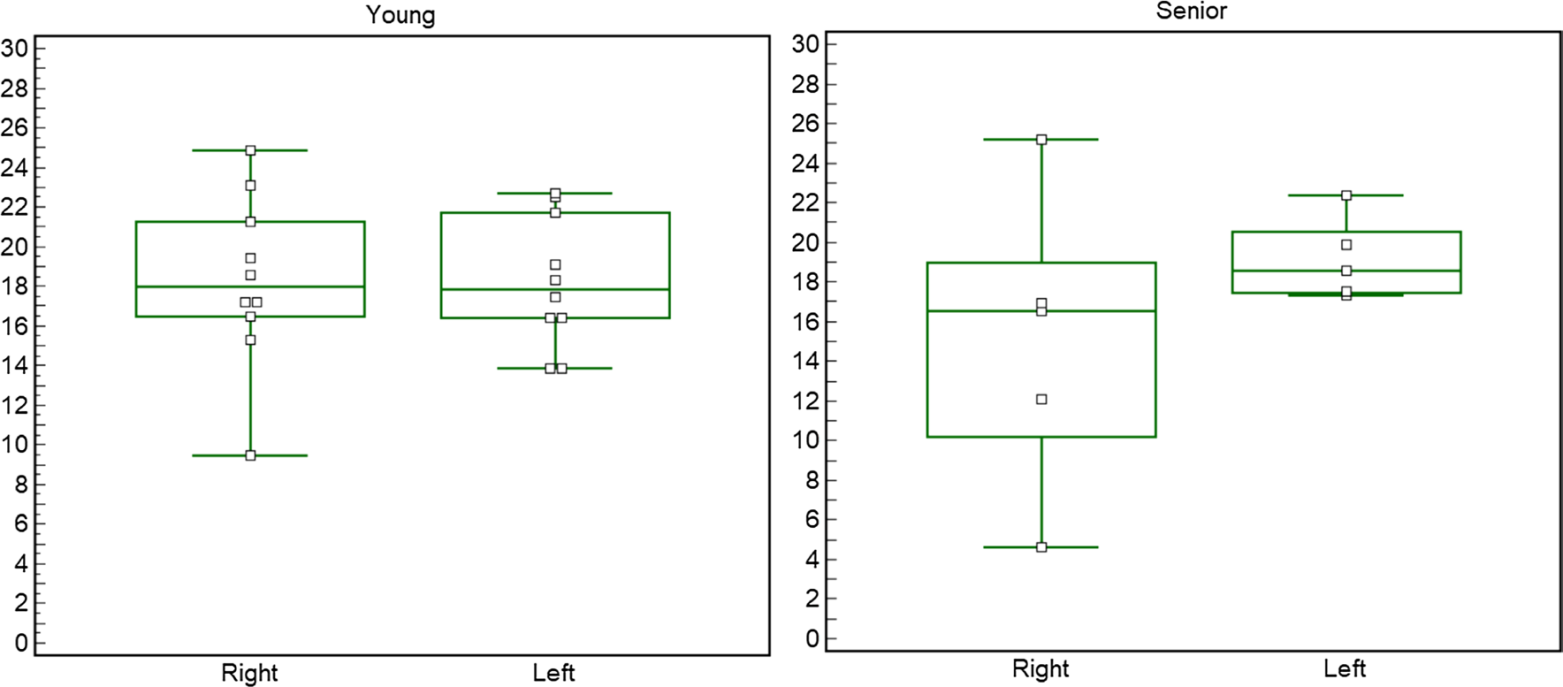
Graph representing the side differences obtained with the Wilcoxon test within the "young" group (ten subjects) and the "senior" group (five subjects). The Wilcoxon test revealed no significant differences in side for aT2 * (young *p* = 1.000; senior *p* = 3.125)

In Table 2, different groups were tested for differences in aT2*. There were no significant differences in sciatic nerve aT2* per side (*p* = 0.38). There were also no significant differences per sex when considering the average of both sides (*p* = 0.92) or when separately assessing right and left (Fig. 2). Finally, there were no significant aT2* differences per age (*p* = 0.50) when averaging sides (*p* = 0.327) and when assessing right and left separately (Fig. 3). CSA (mm^2^) was non-statistically different per side (*p* = 0.87), sex (*p* = 0.18), or age (*p* 0.33).

**Table 2:** aT2* and CSA values per side (right and left) and demographic variables (sex and age)

	Sample size	aT2* (ms)	aT2* SD	*p* value aT2*	CSA (mm^2^)	SD	*p* value CSA
Right	15	17.25	5.54	0.38	48.2	9.0	0.87
Left	15	18.57	2.87		44.9	9.5	
Male	18	17.84	4.95	0.92	48.5	6.5	0.18
Female	12	18.01	3.57		43.8	12.08	
Young	20	18.23	3.73	0.50	44.6	8.4	0.33
Senior	10	17.13	5.62		47.5	6.2	

Total sample size was 30 (two sides for 15 subjects)

Non statistical differences were reported. Bonferroni correction for multiple testing: *α* value 0.05/6 = 0.008

Intra-reader repeatability was excellent, with ICC values, respectively, of 0.95 and 0.9 (both *p* < 0.001) (Table 3) [26]. Inter-reader reproducibility for aT2* measurements was good, with ICC ranging from 0.66 (*p* = 0.030) for the right side and 0.85 (*p* = 0.003) for the left side. The Dice index used for assessing geometrical differences between the drawn ROIs across the nerve boundaries showed were also reasonable, with inter-rater Dice indices between 0.70 and 0.72 and intra-rater values between 0.68 and 0.71.

**Table 3:** Interreader and intrareader agreement

	Right	Left	*p* value	MSE	SD
Reader 1 (**)	17.25 ± 5.54	18.57 ± 2.87	0.157	1.466	5.537
Reader 2 (**)	15.47 ± 5.45	17.81 ± 3.17	0.571	1.466	2.868
DICE (interreader 1 vs 2)	0.70 ± 0.11	0.72 ± 0.12	N/A	N/A	N/A
DICE (intrareader 1 vs 1)	0.71 ± 0,07	0.68 ± 0.11	N/A	N/A	N/A
ICC (interreader 1 vs 2)	0.66 (*p* = 0.029)	0.849 (*p* = 0.007)	N/A	N/A	N/A
ICC (intrareader 1 vs 1)	0.692 (*p* = 0.024)	0.967 (*p* < 0.001)	N/A	N/A	N/A

The Dice coefficient measures the degree of surface overlap of the ROIs drawn by the two operators

*ICC* intraclass correlation coefficient. *SD* standard deviation, *MSE* mean squared error

Measurements of SNR and CNR are reported in Table 4. SNR was significantly higher in Echo 1 compared to Echo 2 image sets. Left side SNR was also slightly higher than right side; however, without reaching statistical significance. CNR was relatively stable per every echo and side.

**Table 4:** Signal-to-noise ratio (SNR) and contrast-to-noise ratio (CNR) measures and standard deviation (SD)

	SNR	SD	CNR	SD
Echo 1 Right	80.39	27.41	0.37	0.14
Echo 2 Right	69.40	20.92	0.39	0.12
Echo 1 Left	99.59	37.26	0.35	0.11
Echo 2 Left	82.78	28.07	0.37	0.13
Echo 1 vs Echo 2	**p = 0.01**		*p* = 0.54	
Right vs Left	*p* = 0.15		*p* = 0.35	

SNR of Echo 1 was significantly higher than Echo 2. Higher SNR was noted on the left side, but did not reach statistical significance. Non significant differences were noted for CNR

## Discussion

In this study, we tested and validated a custom MR sequence capable to generate short-TE images using a vTE approach in a sample of 15 healthy volunteers, aimed at visualizing the short T2* components of the sciatic nerve at the thigh. We demonstrated its technical feasibility and assessed the absence of significant influences in signal variability related to side and demographic features such as age, sex, height, weight, and BMI. Our analysis proved good-to-excellent inter and intra-operator repeatability and reproducibility in aT2* measurements.

Imaging of the nerves is challenging because of their high structural complexity at a small scale. Images from typical MR neurography protocols can resolve the nerve’s fascicles and the epineurium, but not smaller components. In particular, the collagenous connective layers made of short and ultra-short T2 components are not detectable with conventional MRI. Magnetization transfer has proven sensitive to fibrosis [33] and has been suggested in MR neurography protocols [8]. Modern MR sequences such as UTE and ZTE can achieve ultrashort TEs (in the order of microseconds), with data acquisition starting shortly after RF acquisition and ramping the gradient, with k space radial sampling [34–37]. UTE clinical applications are now available for MRI and PET/MRI [38, 39] and promising results on nerves ex-vivo have been described [28]. Nevertheless, UTE are technically challenging and not strictly necessary to visualize tissues rich in collagen. The vTE sequence is less demanding technically and capable to visualize short T2* components (T2* is directly related to T2 relaxation and external field inhomogeneities) with high SNR and resolution.

Our vTE protocol included fat suppression with acquisition of two echoes, the “short” with TE of less than 1 ms, followed by another with TE of about 5 ms. Subtraction of Echo 1 and Echo 2 suppressed the long T2* components and provided images of only-short T2* components. The blurring from the vTE sampling could have had an effect on the signal on the borders with increased signal in the Subtraction image, but this was more pronounced in muscles, especially at the interfaces with tendons and fascia. In nerve, the hyperintensity was diffused and the fibrous peripheral layer (peripheral epineurium) was not evident at this resolution, limiting this effect. Suppression images can be used to visualize the signal change due to the only-short T2* components, as reflected by signal hyperintensity. However quantitative parameters are needed for validation and repeatability. We used a mathematical fit to calculate T2*, and since it was based only on a two-point estimation, we referred to it as “apparent” T2* (aT2*).

Values of aT2* of the sciatic nerve showed small and non-significant difference per side, The signal stability between right and left might allow mono-lateral examinations in pathologies with lack of differences related to laterality [1], halving the scanning time. We also did not find significant dependence of aT2* related to height, weight, or BMI. To remove another possible confounding factor, we investigated differences in nerve CSA. We did not find statistical differences per side, sex or age in our population, even though sex and age correlations with CSA are reported in the literature [40].

We showed good repeatability and reproducibility of the aT2* values (ICC) and the ROI geometry (Dice index) when measured by the same individual. We also found good-to-excellent inter-rater agreement. Measurements of reader 1 were slightly higher than reader 2 though, especially for the right side. We noted also higher standard deviations on the right side, an effect reproduced with both operators. Different levels of experience between the two readers may have influenced these results, but the difference in SNR between the two sides might have played a role, as well. The Dice indexes, expressing ROIs dissimilarity in terms of space, were also similar for intrareader and interreader comparison.

Echo 1 and subtraction presented a slight blurring identified at the skin level and the intramuscular fascia and probably related to the variable sampling. Nevertheless, the CNR of the nerve is similar for both echoes. In addition, some chemical shift artifacts are present, but mainly around the muscles, and the region of the nerve does not seem to be affected. The difference of the two echoes is approximately 4.4 ms, so there should not be an important remaining chemical shift effect. Echo 2 images were normally sampled and more homogeneous, and for this reason, they were used for segmentation. As previously stated, we noted a difference in SNR per side, however SNR measures were affected with great variability (expressed with standard deviations) and no statistical differences per side were found in our population.

This study has some limitations. One of the main drawbacks is the ambiguity of the origin of the aT2* signal. The previous studies have shown that T2* is sensitive both to collagen [25, 27] and myelin [41]. A multi-echo approach would have allowed to estimate the multiple T2* components of T2* relaxation; however, our sequence was originally designed to highlight the short T2* species with the subtraction technique, and only two echoes were acquired. These promising results give the motivation for further experiments using a multi-echo approach, which may determine the origin of the T2* contrast of our sequence**.** Another technical limitation of using short echo times, except the blurring of the first echo, is the lower resolution reachable in a reasonable clinical time [2]. Nonetheless, resolution was sufficient to always depict nerve and internal fascicles, furthermore the sequence is aimed at quantitative imaging and should complement and not substitute conventional MR neurography protocols. Our analysis was focused on a short tract of the sciatic nerve at the mid-thigh. The limited coverage was a compromise between the required resolution and a reasonable scan time. Lesion distribution may be inhomogeneous; for instance, in amyloid neuropathy, most nerve damage is located on the proximal sciatic nerves, rather than on distal tracts [1]. Thus, the vTE sequence could be targeted to a specific nerve tract, based on the pathology to study or the clinical/electrophysiological results. As demonstrated by Chappell et al. [20], the magic angle phenomenon may influence the T2 and T2* measurements in nerves. In our experiments, the sciatic nerves lied mostly parallel to the main magnetic field without anatomical angles or bends, and we used a standard and repeatable positioning to minimize this effect. The study of different anatomic regions and nerves of smaller size should be addressed in the future. Nevertheless, a correlation between histological and imaging findings in patients is not yet available. Further studies correlating imaging and pathological findings in a spectrum of inflammatory, vascular degenerative diseases of the sciatic nerve are warranted.

In conclusion, the vTE sequence is an effective technique that allows highlighting the short T2* components of the sciatic nerves. The signal measured in the nerves appears stable for both thighs and showed no sex, age, or other demographic-related differences. Our study showed that the technique is reproducible and is not significantly intra or inter-operator dependent. Use of the sequence is warranted in future studies investigating neuromuscular disorders.
